# The energy-growth nexus in 3 Latin American countries on the basis of the EKC framework: in the case of Argentina, Brazil, and Chile

**DOI:** 10.1007/s11356-022-24360-3

**Published:** 2022-11-30

**Authors:** Young Kyu Hwang

**Affiliations:** Autonomous University of Madrid, Madrid, Spain

**Keywords:** Environmental Kuznets Curve, CO_2_ emissions, ARDL, Latin America, Economic growth, Energy consumption

## Abstract

In this paper, the effects of economic growth and four different types of energy consumption (oil, natural gas, hydroelectricity, and renewable energy) on environmental quality in terms of carbon dioxide (CO_2_) emissions were examined within the framework of the Environmental Kuznets Curve (EKC) for three Latin American countries, namely, Argentina, Brazil, and Chile, from 1975 to 2018. The autoregressive distributed lag (ARDL) in the form of Error Correction Mechanism (ECM) was used to verify the validity of the EKC hypothesis and the impacts of the variables in the short and the long run alike. Furthermore, the Toda-Yamamoto Granger causality test was carried out to identify the direction of causality between the variables. From ARDL-ECM estimation, the EKC was confirmed (inverted U-shaped curve between income growth and CO_2_ emissions) only in Argentina in the long run but not in Brazil and Chile. Based on the findings, renewable energy can have a great potential in reducing CO_2_ emissions in the future, but this advantage has not been fully exploited yet since a significant negative impact on CO_2_ emissions was only found in Chile. Also, the use of other less carbon-intensive energy sources such as natural gas and hydropower if they could be combined with renewable energy would be of great benefit and contribute to enhancing environmental quality and energy security in the short and the medium term and to successful low-carbon energy transition in the long run in Argentina, Brazil, and Chile.

## Introduction

Growing damages inflicted by climate changes and global warming have been drawing initiatives and collaborations at the global level to challenge them in collective approaches. The greenhouse gases, mainly CO_2_ emissions produced by anthropogenic activities (by burning fossil fuels such as coal, oil, and gas, unsustainable farming practices, and deforestation), are known as the major contributor to global warming (IPCC 2013). The seriousness and severity of the problem are demanding many international initiatives to be taken since 1990 to deal with climate changes and global warming, such as the United Nations Conference on Environment and Development (UNCED) celebrated in Rio de Janeiro in 1992, the Kyoto Protocol in 1997, the World Summit on Sustainable Development in Johannesburg in 2002, the Paris Agreement in 2015, and the recently celebrated COP 26 in Glasgow in 2021 among others (Fuinhas and Marques 2019).

In this context, attentions growing among academia and policymakers advocate needs for new economic growth model in which not only GDP growth but also the improvement of social welfare and the preservation of environment, known as sustainable development, should be encompassed given that the irreversibility of climate changes will accompany enormous economic and social costs without timely addressing. In particular, in Latin American countries, both decoupling economic growth from environmental degradation and achieving sustainable development are particularly challenging in two reasons: their relatively low-income levels (compared to industrialized countries) along with high level of inequality prevailing in the region and their high level of vulnerability to extreme weather events (especially for Caribbean countries).

First, the Latin American and the Caribbean (LAC) mainly consists of middle- and low-income developing countries. Growing economy is a critical issue ahead of them to close the social gap with advanced countries and enhance standards of living by addressing high levels of poverty and income inequality prevailing in this region. However, economic growth is usually accompanied by more energy demand and energy consumption due to economic activities increasing. This implies that the more CO_2_ emissions, the more dependence on polluting and dirty energy sources such as fossil fuels. Accordingly, the country´s environmental quality will be getting worse and worse. Although the LAC region has one of the cleanest electricity mixes in the world thanks to the high share of hydropower in their electricity generation process,^1^ the wide use of it brings a lot of controversies since it can produce a negative impact on local environment during the process of construction of dams coming with destroying ecosystem and reducing biodiversity by displacement of natural habitats and changes in water flows (Cárdenas and Fitzmaurice 2021). Also, hydro energy production is very sensitive to weather conditions (during the dry season, its capacity of electricity generation is seriously affected). Furthermore, the CO_2_ emitted by energy sectors has increased remarkably and it is expecting to keep growing by 132 percent between 2010 and 2050 (Gonzalez Diez et al. 2014).

Second, LAC is highly vulnerable to extreme weather events^2^ even though the region’s contribution to the global GHG emissions account for only 8.3% of the global GHG emissions (Cárdenas et al. 2021). During the last twenty years, the average temperature in the LAC region has increased by 0.1° C per decade in average and the effects of climate change already bring about more frequent forms of natural disasters such as prolonged dry season, a change in the hydrological cycle (change in pattern of precipitation), rising sea levels due to melting glaciers, and consequently a serious risk of floods, especially in Central American and Caribbean countries (IPCC 2013).

Based on our observation and analysis, diversifying energy mix of LAC countries by incorporating more renewable energy sources (not only conventional renewable energy sources like hydropower but also non-conventional renewables such as solar, wind, geothermal, and tidal) can be a good strategy to keep their economy growing without environmental degradation. This is because renewable energy brings about numerous advantages such as energy security in the sense that it contributes to reducing the high dependence on fossil fuels (for oil export countries like Mexico, Colombia, Venezuela, and Ecuador, renewable energy depends less on the volatility of commodity prices in their balance of payments while in the countries importing oil, namely, most of the Caribbean countries, it can provide them with opportunities for reducing dependencies on importing energy by enlarging availability of renewable sources abundant in the region), increasing accessibility to energy for poor households in remote areas, reducing GHG emissions and consequent enhancement of environmental quality, taking attractions of foreign investments on energy sectors, and creating green jobs by investments on renewable energy deployment (Yépez et al. 2016).

In this study, the Environmental Kuznets Curve (EKC) hypothesis during the period of 1975–2018 was verified in 3 Latin American countries: Argentina, Brazil, and Chile, during the period of 1975–2018. They are selected on the basis of the reasons following. On the one hand, they represent the lion’s share of the Latin American economy due to their large size of GDP in the region.^3^ On the other, as they are countries with the highest CO_2_ emissions in the LAC region,^4^ and because they are standing as emerging economies, their carbon emissions are expecting to grow hand in hand with economic growth in the next decade due to an increase in energy demand (in which large share of it is expected to be met by fossil fuel sources). Therefore, analyzing the nexus between economic growth, energy consumption, and CO_2_ emissions in these three countries might provide not only invaluable insight for themselves but also significant implications for other emerging economies. Through the examination of the EKC hypothesis, we tried to determine whether their economic growth is on a sustainable path or not, namely, decoupling their economic growth from environmental deterioration and the impacts of each source of energy consumption on CO_2_ emissions.

The main contributions of our study to existing literature are twofold.

First, to the best of our knowledge, it is the first study that investigates the impacts of energy consumption in a disaggregated manner (oil, natural gas, hydro, and renewable energy) for Argentina, Brazil, and Chile such that it allows us to examine more specifically the impact of each of them on environmental quality in the framework of the EKC. In previous studies of the impacts of energy consumption on environmental quality in Latin American region in the framework of the EKC (Albulescu et al. 2019; Anser et al. 2020; Hanif.^2017^; Jardón et al. 2017; Pablo-Romero and De Jesus 2016; Seri and Fernández 2021; Zilio and Recalde 2011), they are only concerned about renewable and non-renewable energy sources in their research without more on specific identification of diverse energy sources as we approached.

Second, as a novelty, the variables LnAgriland and LnTrade were included in our estimation as proxy for land use change (due to the expansion of agricultural lands and subsequent deforestation) and trade openness, respectively, in these three countries. Why these two variables should be included in our estimation is that they are critically important to determining the level of CO_2_ emissions in addition to energy consumption in these three economies.^5^ However, they are not properly addressed in previous literatures (especially for LnAgriland) on Latin American countries. Thanks to the introduction of land use change and trade openness as our control variables we can take precise estimation of the impact of energy consumption on CO_2_ emissions and minimize omitted variable bias issue at the same time.

The remainder of the paper is organized as follows. “Literature review” provides reviews of the literature survey. “Data and trends of variables” describe the data and the trends of the variables used in our regression models over the period 1975–2018. “Methodology and estimation results” present the methodology, models, and estimation results. “Conclusion and policy implications” conclude with some policy recommendations.

## Literature review

The formal analysis of the nexus between income growth and environmental degradation began with the study of Grossman and Krueger (1991). According to the study, the income growth may affect environmental quality by three different effects: scale, composition, and technological effects (Shahbaz et al. 2019). At the initial stage of economic development, there is an upsurge in energy consumption and resource use due to a rise in economic activities known as scale effects which lead to increasing carbon emissions and environmental degradation. As income grows, economy meets a series of structural change known as composition effect. During the initial stage of industrialization, the economy shifts from agricultural sector to industrial sector and brings about accelerated economic growth together with a rapid environmental deterioration due to energy-intensive polluting industries which gives rise to a growing concern about environmental quality. Therefore, polluting industries are gradually replaced by those with clean energy technology and knowledge-intensive service sector grown up at the later stage of industrialization (Shahbaz et al. 2019). Consequently, CO_2_ emission levels begin to decrease, and after reaching a certain threshold income (turning point of EKC), the relation between economic growth and pollution level turns into negative. At this moment, lavish investments in innovative technology and green infrastructure are made to prevent environmental degradation which leads to further improvement in environmental quality. This is known as a technological effect.

Regarding validity of the EKC, there has been no consensus among the researchers. The estimation results differ largely across countries or regions, the time period considered, and econometric techniques used. Some researchers found evidence of the EKC relationship in both developed and in developing countries^6^ (Akram et al. (2020) for selected South Asian, Asian, and most of the African countries; Cantos and Lorente (2011) for Spain; Destek and Sinha (2020) for OECD countries; Khan et al. (2021) for USA; Narayan and Narayan (2010) for the Middle Eastern and South Asian countries; Sarkodie and Strezov (2018) for Australia; Shah et al. (2021) for Western Asia and North African countries in case of ecological footprint as a dependent variable; Shahbaz et al. (2019) for Vietnam in the long run) while others could not verify the EKC relationship (Dogan et al. (2020) for Brazil, Russia, India, China, South Africa, and Turkey; Liu et al. (2017) for Indonesia Malaysia, the Philippines, and Thailand).

In relation to the LAC region, the presence of the EKC relationship is ambiguous. Some authors found only partial validation of the EKC. Albulescu et al. (2019) investigated the relationship between income, environmental deterioration, and FDI for 14 LAC countries from 1980 to 2010 using panel quantile regression but they could not verify the EKC hypothesis in lower-income countries. Seri and Fernández (2021) also confirm partial validation of the EKC hypothesis in the region. They studied the nexus between income and per capita carbon dioxide emissions in 21 LAC economies from 1960 to 2017 using the ARDL bounds testing and the UECM and found that the EKC hypothesis is validated only in a few of LAC countries.

No evidence of the EKC hypothesis in the LAC countries was also confirmed in several studies. Zilio and Recalde (2011) analyzed the nexus between economic growth and energy consumption for 21 LAC countries from 1970 to 2007 by using cointegration approach and they could not verify the EKC hypothesis because of the absence of long-run relationship between the variables. Pablo-Romero and De Jesus (2016) studied the nexus between economic growth and energy consumption in 22 LAC countries from 1990 to 2011 by using absolute energy consumption as a proxy for environmental pressure, but they found no evidence of the EKC hypothesis in the LAC region. Jardón et al. (2017) examined the EKC relationship in a set of 20 LAC countries from 1971 to 2011 using the FMOLS and the DOLS, and they could not verify existence of the EKC due to a lack of long-run equilibrium relationship between the variables.

However, Hanif (2017) could verify the EKC hypothesis by investigating the EKC relationship in a panel of 20 middle and lower-middle income countries from 1990 to 2015 using the system GMM with a two-step estimator. He also found that renewable energy consumption contributes to meeting growing energy demand and reducing the trade deficit in the LAC region. In the study of Anser et al. (2020), the EKC hypothesis was examined in 16 middle and lower-middle income countries in the LAC region using panel data analysis. They chose fossil fuel consumption, renewable energy use, and industrial growth index as variables in their regression model and applied a two-step GMM robust estimator as econometric technique. They confirm the existence of the EKC and found that both consumption of fossil fuels and industrial growth contribute to an increase in pollution levels (CO_2_ emissions) in the LAC region.

There are few studies that investigated the impacts of trade openness and/or agricultural lands in addition to energy consumption on CO_2_ emissions under the framework of the EKC. For trade openness, Ozatac et al. (2017) investigated (besides energy consumption, urbanization, and financial development) its impacts on environmental quality for Turkey from 1960 to 2013. They used ARDL estimation and Granger causality test and found positive impact of trade on CO_2_ emissions (thus environmental deterioration) both in the short and the long run and unidirectional causality from trade openness to carbon emissions. For agricultural lands, Gokmenoglu and Taspinar (2018) examined the agriculture-induced EKC for Pakistan from 1971 to 2014 by using FMOLS and they found that agricultural development leads to increased CO_2_ emissions (although the magnitude of its long-run coefficient is significantly lower than that of GDP) and they also confirmed EKC hypothesis for Pakistan. Gokmenoglu et al. (2019) carried out investigation on the long-run equilibrium relationship between CO_2_ emissions, income growth, energy consumption, and agriculture in the framework of agriculture-induced EKC for China during the period from 1971 to 2014. The results of their ARDL estimation suggest that agricultural development contributes to increasing CO_2_ emissions both in the short and long run and the verification of EKC hypothesis in China.

## Data and trends of variables

### Data

In our investigation, the relationship between energy consumption, economic growth, and environmental quality in the framework of the EKC was examined in three Latin American countries, namely, Argentina, Brazil, and Chile, over the period from 1975 to 2018. For this purpose, nine variables were used in total, namely, per capita carbon dioxide emissions, per capita GDP and squared GDP per capita, and per capita energy consumption (oil, gas, hydroelectricity, and renewable energy), in addition to agricultural land and trade openness as control variables in our regression model (see Table 1). The reason behind the selection of these four types of energy consumption is that they constitute four main energy sources with high shares in final energy consumption in Argentina, Brazil, and Chile (although the share of each energy source varies from one country to another). As we can see in Table 2, we have 44 observations for each country. In all cases, we can observe a relatively high variability of squared GDP per capita, natural gas consumption, and renewable energy consumption in comparison with other variables.

**Table 1:** Variable description

Variable	Definition	Source
LnCO2pc	Carbon dioxide emissions per capita (million tons of carbon dioxide per capita)	British Petroleum (BP)
LnGDPpc	Per capita GDP (constant 2015 US dollars)	World Development Indicators (World Bank)
LnGDPpc2	Squared per capita GDP (constant 2015 US dollars)	World Development Indicators (World Bank)
LnOCpc	Oil consumption per capita (kWh per capita)	British Petroleum (BP)
LnNGaspc	Natural gas consumption (kWh per capita)	British Petroleum (BP)
LnHydropc	Hydroelectricity consumption (kWh per capita)	British Petroleum (BP)
LnRECpc	Renewable energy consumption (kWh per capita)	British Petroleum (BP)
LnAgriland	Agricultural land (% of land area)	World Development Indicators (World Bank)
LnTrade	Trade (% of GDP)	World Development Indicators (World Bank)

In case of different sources of energy consumption (oil, natural gas, hydro, and renewable energy), energy units were converted from exajoules to kWh

**Table 2:** Descriptive statistics

Variable	Obs	Mean	Std. Dev	Min	Max
Argentina					
LnCO2pc	44	− 12.48948	0.1004198	− 12.67677	− 12.31398
LnGDPpc	44	9.283141	0.1587217	9.00568	9.561016
LnGDPpc2	44	86.20132	2.957292	81.10227	91.41303
LnOCpc	44	9.014026	0.1585661	8.728825	9.362474
LnNGaspc	44	8.873877	0.3799986	8.12676	9.311071
LnHydropc	44	7.535164	0.4410069	6.273217	8.039916
LnRECpc	44	3.596268	1.606436	1.669497	6.378991
LnAgriland	44	3.890297	0.0632381	3.840425	3.9989
LnTrade	44	3.078054	0.4068498	2.446311	3.731765
Brazil					
LnCO2pc	44	− 13.30562	0.1950054	− 13.5704	− 12.89715
LnGDPpc	44	8.842032	0.1573781	8.550295	9.132116
LnGDPpc2	44	78.20573	2.794005	73.10755	83.39555
LnOCpc	44	8.618496	0.1408026	8.391888	8.915105
LnNGaspc	44	5.980633	1.183036	3.667276	7.649634
LnHydropc	44	8.294996	0.2605114	7.535204	8.634068
LnRECpc	44	6.235082	0.9773826	3.453038	7.775524
LnAgriland	44	3.319312	0.029484	3.213624	3.365696
LnTrade	44	3.030947	0.2209938	2.666595	3.390414
Chile					
LnCO2pc	44	− 12.72336	0.413913	− 13.32722	− 12.16887
LnGDPpc	44	8.908135	0.4467449	8.176196	9.539718
LnGDPpc2	44	79.54992	7.944941	66.85017	91.00621
LnOCpc	44	8.965935	0.3511062	8.412502	9.499393
LnNGaspc	44	7.415583	0.8163221	6.258295	8.679123
LnHydropc	44	7.950681	0.2754387	7.4237	8.436293
LnRECpc	44	5.264094	1.413834	2.593349	7.770879
LnAgriland	44	3.064898	0.0355924	3.008386	3.141253
LnTrade	44	4.042415	0.1724924	3.685504	4.39185

### Trends of variables

**Fig. 1:**
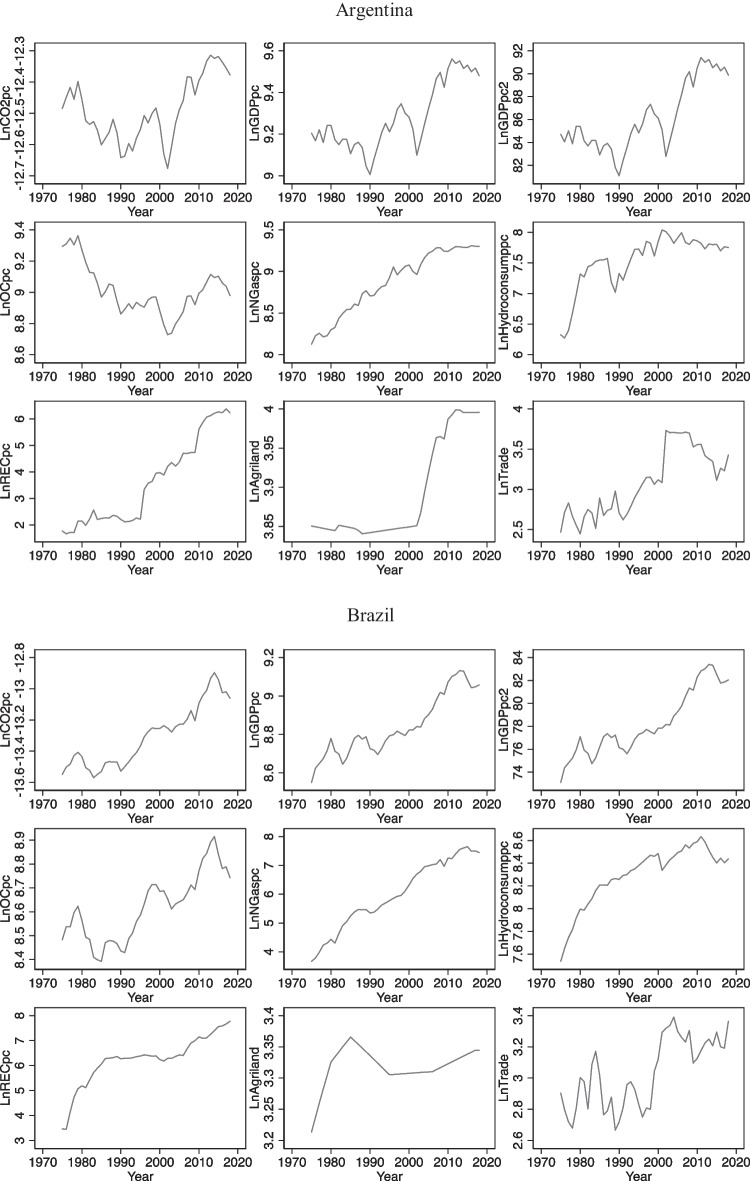

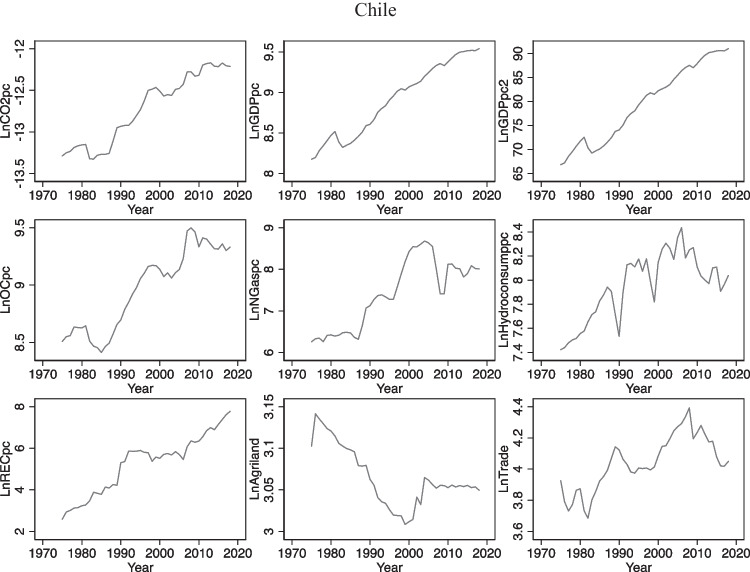
Evolution of the variables in levels over the period 1975–2018

In Fig. 1, we can observe general trends of each variable in 3 countries. In the case of Brazil and Chile, in general, we can see an upward trend of per capita CO_2_ emissions over the period of study while in Argentina there is a decline in per capita CO_2_ emissions up to the beginning of the 2000s, and then, it increases considerably with some fluctuations. In relation to per capita GDP, all 3 countries have improved their income level over the period, but in the case of Argentina, large fluctuations were observed while in Brazil and Chile upward trends with much less fluctuations were observed. This explains in some way high instability of Argentinian economy and its vulnerability to several external shocks compared to other 2 economies during the period. Regarding energy consumption, upward trends of oil consumption were found in Brazil and Chile while in Argentina the level of oil consumption in 2018 reduced with respect to that of 1975. Also, we can observe that consumption of other 3 types of energy, namely, natural gas, hydroelectricity, and renewable energy, has been increasing over time in Argentina, Brazil, and Chile. Apart from the specific paths followed by each variable, none of them seems to be stationary at levels and they have unit root problem (mean and variance change and do not remain constant over time) as we can see in Fig. 1.

## Methodology and estimation results

In this section, we proceed to estimate the impacts of different energy consumption and economic growth on environmental quality in the framework of the EKC in Argentina, Brazil, and Chile. To this point, 3 models were estimated using the following equations:

1 $$Ln{CO}_{2}=f(Ln{GDP}_{pc},{LnGDP}_{pc}^{2}, {LnOC}_{pc}, {LnNGas}_{pc},{ LnHydro}_{pc }, {LnREC}_{pc})$$

2 $$Ln{CO}_{2}=f(Ln{GDP}_{pc},{LnGDP}_{pc}^{2}, {LnOC}_{pc}, {LnNGas}_{pc},{ LnHydro}_{pc }, {LnREC}_{pc}, LnAgriland)$$

3 $$Ln{CO}_{2}=f(Ln{GDP}_{pc},{LnGDP}_{pc}^{2}, {LnOC}_{pc}, {LnNGas}_{pc},{ LnHydro}_{pc }, {LnREC}_{pc}, LnAgriland, Ln Trade)$$

In model 1, we include only GDP, squared GDP, and different sources of energy consumption in our regression model while in models 2 and 3 we added agricultural land and trade, respectively, as our control variables to test the robustness of our estimation results.

Our estimation process consists of six steps: unit root test, cointegration bounds test, ARDL-ECM estimation, postestimation tests, CUSUM and CUSUMSQ tests, and lastly the Toda-Yamamoto Granger causality test.

### Unit root test

To test formally the stationarity and the presence of unit root problem, we performed the Zivot-Andrews test (Zivot and Andrews 2002). The advantage of this test is that it allows to check unit root in the presence of one structural break compared to traditional unit root tests such as the Augmented Dickey-Fuller and the Phillips-Perron tests (Dickey and Fuller 1979; Phillips and Perron 1988) which do not consider structural break when testing the unit root hypothesis. As we can see in Table 3, all variables are stationary at first differences without exception, namely, all of them are integrated of order 1 or $I(1)$ (in the case of LnTrade and LnRECpc in Argentina and Brazil, they are even integrated of order 0 or $I(0)$) and none of them seems to have an order of integration greater than 1. The condition mentioned above is very important before performing bounds test and the ARDL model since variables integrated of order 2 or superior are not allowed (Menegaki, 2020).

**Table 3:** Unit root tests

Variables	Level		First difference		Order of integration
	$t$-statistic	Structural break	$t$-statistic	Structural break	
Argentina					
LnCO2pc	− 3.941	1989	− 5.996***	2003	$I\left(1\right)$
LnGDPpc	− 2.775	1985	− 6.349***	2003	$I\left(1\right)$
LnGDPpc2	− 2.775	2005	− 6.372***	2003	$I\left(1\right)$
LnOCpc	− 3.357	1989	− 5.419**	2004	$I\left(1\right)$
LnNGaspc	− 2.907	1995	− 6.599***	1985	$I\left(1\right)$
LnHydropc	− 2.834	1982	− 8.312***	1990	$I\left(1\right)$
LnRECpc	− 4.543	1996	− 7.448***	1996	$I\left(1\right)$
LnAgriland	− 2.976	2004	− 6.739***	2003	$I\left(1\right)$
LnTrade	− 6.649***	2002	− 8.236***	2004	$I(0)/I(1)$
Brazil					
LnCO2pc	− 4.096	1988	− 6.455***	2010	$I\left(1\right)$
LnGDPpc	− 3.562	1990	− 5.686***	1984	$I\left(1\right)$
LnGDPpc2	− 3.528	1990	− 5.638***	1984	$I\left(1\right)$
LnOCpc	− 3.037	1983	− 5.230**	2010	$I\left(1\right)$
LnNGaspc	− 3.075	2001	− 7.215***	1999	$I\left(1\right)$
LnHydropc	− 3.762	2010	− 6.798***	2003	$I\left(1\right)$
LnRECpc	− 6.588***	2007	− 7.053***	1990	$I(0)/I(1)$
LnAgriland	− 4.465	1997	− 6.481***	1986	$I\left(1\right)$
LnTrade	− 4.231	1999	− 5.855***	1990	$I\left(1\right)$
Chile					
LnCO2pc	− 3.602	1988	− 5.304**	1984	$I\left(1\right)$
LnGDPpc	− 4.289	1991	− 6.926***	1984	$I\left(1\right)$
LnGDPpc2	− 4.252	1991	− 6.757***	1984	$I\left(1\right)$
LnOCpc	− 2.899	2006	− 5.570**	1986	$I\left(1\right)$
LnNGaspc	− 3.779	2007	− 6.011***	2005	$I\left(1\right)$
LnHydropc	− 3.972	2005	− 7.283***	1991	$I\left(1\right)$
LnRECpc	− 3.365	1998	− 8.790***	1993	$I\left(1\right)$
LnAgriland	− 3.951	2002	− 6.263***	2001	$I\left(1\right)$
LnTrade	− 3.510	2004	− 6.438***	2009	$I(1)$

Zivot-Andrews unit root test was carried out under the specification of intercept and trend

^*^, **, ***10%, 5%, and 1% statistical significance level, respectively

### Bounds cointegration test

The next step after testing order of integration, stationarity, and unit root problem consists of examining cointegrations among the variables by means of the ARDL bounds test. For our purpose, the Pesaran, Shin, and Smith bounds test (2001) was used. In this test, the null hypothesis of ${H}_{0 }= {\alpha }_{0}={\alpha }_{1}={\alpha }_{2}={\alpha }_{3}\dots ..={\alpha }_{n}=0$ is tested against the alternative one of ${H}_{0 }\ne {\alpha }_{0}\ne {\alpha }_{1}\ne {\alpha }_{2}\ne {\alpha }_{3}\dots ..\ne {\alpha }_{n}\ne 0$. If we fail to reject the null hypothesis at the given statistical level, there is no cointegration among the variables. Otherwise, there is a long-run equilibrium relationship (cointegration) among the variables if the opposite is true.

As we can see in Table 4, evidence of cointegration relationship between dependent and independent variables in our regression (CO_2_, GDP, squared GDP, different sources of energy consumption, agricultural land, and trade openness) is very strong in Argentina, Brazil, and Chile. The $F$-statistics exceed upper-bound limit at 1% significance level except for model 1 for Chile where cointegration is found at 5% significance level. Once the long-run relationships among the variables were confirmed, we proceed to estimate the ARDL model together with the Error Correction Mechanism (ECM).

**Table 4:** Bounds test

Models	Lag order selection	Critical value bounds			$F$-statistic	Decision
		10%	5%	1%		
Argentina						
1	ARDL(1,2,2,0,2,0,1)	2.321–3.697	2.796–4.363	3.944–5.957	16.966***	Cointegration
2	ARDL(2,2,2,0,1,0,2,0)	2.229–3.645	2.681–4.296	3.779–5.868	32.176***	Cointegration
3	ARDL(1,1,1,0,1,1,1,0,1)	2.167–3.554	2.590–4.164	3.613–5.630	27.357***	Cointegration
Brazil						
1	ARDL(1,1,1,0,2,1,2)	2.321–3.697	2.796–4.363	3.944–5.957	24.088***	Cointegration
2	ARDL(2,1,1,0,0,0,2,1)	2.242–3.620	2.691–4.257	3.774–5.785	22.557***	Cointegration
3	ARDL(1,1,1,0,0,0,1,0,1)	2.179–3.530	2.599–4.126	3.608–5.553	23.212***	Cointegration
Chile						
1	ARDL(1,2,2,1,2,2,2)	2.292–3.747	2.774–4.442	3.950–6.124	4.530**	Cointegration
2	ARDL(2,2,2,1,1,2,0,1)	2.216–3.670	2.672–4.336	3.784–5.951	8.536***	Cointegration
3	ARDL(1,1,0,1,1,0,0,1,0)	2.179–3.530	2.599–4.126	3.608–5.553	7.598***	Cointegration

Regarding optimal lag selection, we used the Bayesian Information Criterion (BIC) and set as maximum lags of 2 for models 1 and 2, respectively, and maximum lag of 1 for model 3 to avoid multicollinearity issue; we also carried out cointegration tests by using $t$-statistics and we found that the calculated $t$-statistics exceeded the upper-bounds critical values at 5% statistical significance level in all cases confirming the cointegration relationship among the variables

^*^, **, ***10%, 5%, and 1% statistical significance level, respectively

### ARDL-ECM estimation

To estimate the short-run and long-run elasticities of variables, the ARDL-ECM was used. The ARDL method was first developed by Pesaran and Shin (1995), and since then, it is widely used in the field of energy economics. Compared to another econometric methods, ARDL offers several advantages (Menegaki 2020). First, ARDL is easy to implement, and it allows to estimate the short-run and the long-run elasticities simultaneously in the single structure equation (Bayer and Hanck 2013). Second, it allows to estimate parameters in the framework of optimal lag criteria which eliminates serial correlation issue (Ali et al. 2017) and it provides unbiased estimator even if the model suffers from endogeneity problem (Harris and Sollis 2003). Third, the ARDL model is highly flexible because it can be estimated regardless of integration order ($I(0)$, $I(1)$, or mix of them) as long as the variables do not have an order of integration greater than one.

Three ARDL models used in our estimation can be written as follows:

4 $${LnCO}_{2pc}={\alpha }_{1}+\sum\nolimits_{i=1}^{l}{\beta }_{11}{LnCO}_{2pc,t-i}+\sum\nolimits_{i=1}^{m}{{\beta }_{12}LnGDP}_{pc,t-i}+\sum\nolimits_{i=1}^{n}{{\beta }_{13}LnGDP}_{pc2,t-i}+\sum\nolimits_{i=1}^{o}{{\beta }_{14}LnOC}_{pc,t-i}+\sum\nolimits_{i=1}^{p}{{\beta }_{15}LnNGas}_{pc,t-i}+\sum\nolimits_{i=1}^{q}{{\beta }_{16}LnHydro}_{pc,t-i}+\sum\nolimits_{i=1}^{r}{{\beta }_{17}LnREC}_{pc,t-i}+{\mu }_{1t}$$

5 $${LnCO}_{2pc}={\alpha }_{2}+\sum\nolimits_{i=1}^{l}{\beta }_{21}{LnCO}_{2pc,t-i}+\sum\nolimits_{i=1}^{m}{{\beta }_{22}LnGDP}_{pc,t-i}+\sum\nolimits_{i=1}^{n}{{\beta }_{23}LnGDP}_{pc2,t-i}+\sum\nolimits_{i=1}^{o}{{\beta }_{24}LnOC}_{pc,t-i}+\sum\nolimits_{i=1}^{p}{{\beta }_{25}LnNGas}_{pc,t-i}+\sum\nolimits_{i=1}^{q}{{\beta }_{26}LnHydro}_{pc,t-i}+\sum\nolimits_{i=1}^{r}{{\beta }_{27}LnREC}_{pc,t-i}+\sum\nolimits_{i=1}^{s}{{\beta }_{28}LnAgriland}_{,t-i}+{\mu }_{2t}$$

6 $${LnCO}_{2pc}={\alpha }_{3}+\sum\nolimits_{i=1}^{l}{\beta }_{31}{LnCO}_{2pc,t-i}+\sum\nolimits_{i=1}^{m}{{\beta }_{32}LnGDP}_{pc,t-i}+\sum\nolimits_{i=1}^{n}{{\beta }_{33}LnGDP}_{pc2,t-i}+\sum\nolimits_{i=1}^{o}{{\beta }_{34}LnOC}_{pc,t-i}+\sum\nolimits_{i=1}^{p}{{\beta }_{35}LnNGas}_{pc,t-i}+\sum\nolimits_{i=1}^{q}{{\beta }_{36}LnHydro}_{pc,t-i}+\sum\nolimits_{i=1}^{r}{{\beta }_{37}LnREC}_{pc,t-i}+\sum\nolimits_{i=1}^{s}{{\beta }_{38}LnAgriland}_{,t-i}+\sum\nolimits_{i=1}^{t}{{\beta }_{39}LnTrade}_{,t-i}+{\mu }_{3t}$$

${\alpha }_{k}$ represents intercept, ${\beta }_{k}$ represents the coefficient of parameter, and ${\mu }_{t}$ stands for error term.

To assess the speed of adjustment, namely, how fast the economy recovers from the shock and back to equilibrium and to estimate the short- and long-run elasticities (Fuinhas and Marques 2019), we incorporate the ECT in our ARDL models:

7 $$\Delta {LnCO}_{2pc}={\sigma }_{1}+\sum\nolimits_{i=1}^{l}{\varphi }_{11}{\Delta LnCO}_{2pc,t-i}+\sum_{i=1}^{m}{{\varphi }_{12}\Delta LnGDP}_{pc,t-i}+\sum\nolimits_{i=1}^{n}{{\varphi }_{13}\Delta LnGDP}_{pc2,t-i}+\sum\nolimits_{i=1}^{o}{{\varphi }_{14}\Delta LnOC}_{pc,t-i}+\sum\nolimits_{i=1}^{p}{{\varphi }_{15}\Delta LnNGas}_{pc,t-i}+\sum\nolimits{i=1}^{q}{{\varphi }_{16}\Delta LnHydro}_{pc,t-i}+\sum\nolimits_{i=1}^{r}{{\Delta \varphi }_{17}LnREC}_{pc,t-i}+{\theta }_{11}{LnCO}_{2pc,t-1}+{\theta }_{12}{LnGDP}_{pc,t-1}+{\theta }_{13}{LnGDP}_{pc2,t-1}+{\theta }_{14}{LnOC}_{pc,t-1}+{\theta }_{15}{LnNGas}_{pc,t-1}+{\theta }_{16}{LnHydro}_{pc,t-1}+{\theta }_{17}{LnREC}_{pc,t-1}+{\varepsilon }_{1t}$$

8 $$\Delta {LnCO}_{2pc}={\sigma }_{2}+\sum\nolimits_{i=1}^{l}{\varphi }_{21}{\Delta LnCO}_{2pc,t-i}+\sum\nolimits_{i=1}^{m}{{\varphi }_{22}\Delta LnGDP}_{pc,t-i}+\sum\nolimits_{i=1}^{n}{{\varphi }_{23}\Delta LnGDP}_{pc2,t-i}+\sum\nolimits_{i=1}^{o}{{\varphi }_{24}\Delta LnOC}_{pc,t-i}+\sum\nolimits_{i=1}^{p}{{\varphi }_{25}\Delta LnNGas}_{pc,t-i}+\sum\nolimits_{i=1}^{q}{{\varphi }_{26}\Delta LnHydro}_{pc,t-i}+\sum\nolimits_{i=1}^{r}{{\Delta \varphi }_{27}LnREC}_{pc,t-i}++\sum\nolimits_{i=1}^{s}{{\Delta \varphi }_{28}LnAgriland}_{,t-i}+{\theta }_{21}{LnCO}_{2pc,t-1}+{\theta }_{22}{LnGDP}_{pc,t-1}+{\theta }_{23}{LnGDP}_{pc2,t-1}+{\theta }_{24}{LnOC}_{pc,t-1}+{\theta }_{25}{LnNGas}_{pc,t-1}+{\theta }_{26}{LnHydro}_{pc,t-1}+{\theta }_{27}{LnREC}_{pc,t-1}+{\theta }_{28}{LnAgriland}_{,t-1}+{\varepsilon }_{2t}$$

9 $$\Delta {LnCO}_{2pc}={\sigma }_{3}+\sum\nolimits_{i=1}^{l}{\varphi }_{31}{\Delta LnCO}_{2pc,t-i}+\sum\nolimits_{i=1}^{m}{{\varphi }_{32}\Delta LnGDP}_{pc,t-i}+\sum\nolimits_{i=1}^{n}{{\varphi }_{33}\Delta LnGDP}_{pc2,t-i}+\sum\nolimits_{i=1}^{o}{{\varphi }_{34}\Delta LnOC}_{pc,t-i}+\sum\nolimits_{i=1}^{p}{{\varphi }_{35}\Delta LnNGas}_{pc,t-i}+\sum\nolimits_{i=1}^{q}{{\varphi }_{36}\Delta LnHydro}_{pc,t-i}+\sum\nolimits_{i=1}^{r}{{\Delta \varphi }_{37}LnREC}_{pc,t-i}++\sum\nolimits_{i=1}^{s}{{\Delta \varphi }_{38}LnAgriland}_{,t-i}+\sum\nolimits_{i=1}^{t}{{\Delta \varphi }_{38}LnTrade}_{,t-i}+{\theta }_{31}{LnCO}_{2pc,t-1}+{\theta }_{32}{LnGDP}_{pc,t-1}+{\theta }_{33}{LnGDP}_{pc2,t-1}+{\theta }_{34}{LnOC}_{pc,t-1}+{\theta }_{35}{LnNGas}_{pc,t-1}+{\theta }_{36}{LnHydro}_{pc,t-1}+{\theta }_{37}{LnREC}_{pc,t-1}+{\theta }_{38}{LnAgriland}_{,t-1}+{\theta }_{39}{LnTrade}_{,t-1}+{\varepsilon }_{3t}$$

$\Delta$ denotes the first difference, ${\sigma }_{k}$ denotes intercept, ${\varphi }_{k},{\theta }_{k}$ represent the coefficients of parameter, and ${\varepsilon }_{t}$ represent error term. The ECT (error correction term) lagged once is given by the coefficient of LnCO_2_pc (− 1), namely, ${\theta }_{11}$, ${\theta }_{21}$, and ${\theta }_{31}$, for models 1, 2, and 3, respectively.

The ARDL estimation results are presented in Table 5. For each country, three different ARDL-ECM models were estimated. In model 1, we include only LnCO_2_pc, LnGDPpc, LnGDPpc2, and four different sources of energy consumption (see Eq. 7) while in model 2 and 3 we included LnAgriland only and included both LnAgriland and LnTrade, respectively, to reduce omitted variable bias issue and to examine the robustness of our regression model (see Eqs. 8 and 9). The specific reason for including LnAgriland as our control variable is that change in land use caused by expanding agricultural land (due to the heavy dependence of Latin American economies on commodities export) is an important factor to carbon dioxide emissions in Latin American region as it leads to deforestation and consequently reduces the capacity of nature to capture and store CO_2_. Furthermore, we also included LnTrade as our control variable since it might impact importantly on environmental quality and carbon dioxide emissions. The impacts of trade on environmental quality can be mixed. On the one hand, it might contribute to improving environmental quality through the import of clean energy technology from industrialized countries. Otherwise, it might lead to increasing CO_2_ emissions via export activities in case of increased energy consumption required to produce more goods or services mainly comes from dirty energy sources such as fossil fuels.

- Argentina

**Table 5:** ARDL-ECM estimation results

Variable	Coefficient		$t$-statistic			Coefficient		$t$-statistic			Coefficient		$t$-statistic		
Argentina															
	Model 1 (1,2,2,0,2,0,1)					Model 2 (2,2,2,0,1,0,2,0)					Model 3 (1,1,1,0,1,1,1,0,1)				
ECT(− 1)	− 0.7683351*** (0.0866933)		− 8.86			− 0.8787117*** (0.0686229)		− 12.80			− 0.7900327*** (0.0646232)		− 12.23		
Long-run estimations															
LnGDPpc(− 1)	7.436119** (3.536188)		2.10			13.30518*** (2.987095)		4.45			5.535668* (3.065336)		1.81		
LnGDPpc2(− 1)	− 0.4069416** (0.1924509)		− 2.11			− 0.7263499*** (0.1634268)		− 4.44			− 0.3065657* (0.1662582)		− 1.84		
LnOCpc(− 1)	0.7401964*** (0.1115497)		6.64			0.7152715*** (0.0775447)		9.22			0.7873526*** (0.0738874)		10.66		
LnNGaspc(− 1)	0.2944676*** (0.0613933)		4.80			0.271748*** (0.0451976)		6.01			0.2927168*** (0.0374369)		7.82		
LnHydropc(− 1)	− 0.0329108 (0.0199846)		− 1.65			− 0.0161355 (0.0133974)		− 1.20			− 0.0124894 (0.0120663)		− 1.04		
LnRECpc(− 1)	0.019054** (0.0072742)		2.62			0.0085822 (0.0056672)		1.51			− 0.0022279 (0.0050666)		− 0.44		
LnAgriland(− 1)	–		–			0.5246465*** (0.1381332)		3.80			0.3350252** (0.130934)		2.56		
LnTrade(− 1)	–		–			–		–			0.0532344*** (0.0136882)		3.89		
Short-run estimations															
DLnGDPpc	− 0.6005088 (2.817552)		− 0.21			4.012602 (2.523129)		1.59			2.746488 (2.178388)		1.26		
DLnGDPpc(− 1)	− 6.326025** (2.762895)		− 2.29			− 8.378995*** (2.177885)		− 3.85			–		–		
DLnGDPpc2	0.0376745 (0.1525642)		0.25			− 0.2171816 (0.1377169)		− 1.58			− 0.1490937 (0.1183577)		− 1.26		
DLnGDPpc2(− 1)	0.3454021** (0.1494719)		2.31			0.4530866*** (0.1179829)		3.84			–		–		
DLnOCpc	0.5687189*** (0.0826463)		6.88			0.6285174*** (0.0630712)		9.97			0.6220343*** (0.0576803)		10.78		
DLnNGaspc	0.3273578*** (0.0486701)		6.73			0.2783659*** (0.0427926)		6.51			0.3283875*** (0.041295)		7.95		
DlnNGaspc(− 1)	− 0.0183502 (0.0457669)		− 0.40			–		–			–		–		
DLnHydropc	− 0.0252865 (0.0156832)		− 1.61			− 0.0141785 (0.0118523)		− 1.20			− 0.0058453 (0.0158058)		− 0.37		
DLnRECpc	0.0173852 (0.0115891)		1.50			0.0132259 (0.0084847)		1.56			0.0047392 (0.0067088)		0.71		
DLnRECpc(− 1)	–		–			− 0.0087892 (0.0089028)		− 0.99			–		–		
DLnAgriland	–		–			0.461013*** (0.1241534)		3.71			0.2646809** (0.1083225)		2.44		
DLnTrade	–		–			–		–			0.0209229 (0.0135609)		1.54		
Constant	− 42.68296*** (12.64309)		− 3.88			− 73.9903*** (12.53399)		− 5.90			− 38.32895*** (12.15516)		− 3.15		
Brazil															
	Model 1 (1,1,1,0,2,1,2)					Model 2 (2,1,1,0,0,0,2,1)					Model 3 (1,1,1,0,0,0,1,0,1)				
ECT(− 1)	− 0.9160742*** (0.1001981)			− 9.14		− 0.9911566*** (0.1149028)			− 8.63		− 0.8885256*** (0.1089206)			− 8.16	
Long-run estimations															
LnGDPpc(− 1)	− 9.317779** (3.816529)			− 2.44		− 6.015536* (3.468424)			− 1.73		− 6.096905 (3.586267)			− 1.70	
LnGDPpc2(− 1)	0.5297702** (0.2122999)			2.50		0.3473731* (0.1945302)			1.79		0.3495479* (0.1989248)			1.76	
LnOCpc(− 1)	0.8409074*** (0.0398518)			21.10		0.7787191*** (0.0580378)			13.42		0.8463142*** (0.0544476)			15.54	
LnNGaspc(− 1)	0.0761091*** (0.0097743)			7.79		0.0686745*** (0.0081382)			8.44		0.0654339*** (0.0173359)			3.77	
LnHydropc(− 1)	− 0.1408244*** (0.0399231)			− 3.53		− 0.1411248*** (0.0357902)			− 3.94		− 0.1270753*** (0.0362592)			− 3.50	
LnRECpc(− 1)	0.0120785 (0.0091891)			1.31		0.025626** (0.0123058)			2.08		0.022739 (0.0150212)			1.51	
LnAgriland(− 1)	–			–		− 0.151479 (0.1863728)			− 0.81		− 0.022609 (0.2288408)			− 0.10	
LnTrade(− 1)	–			–		–			–		0.0049995 (0.0337135)			0.15	
Short-run estimations															
DLnGDPpc	− 1.52678 (5.75949)			− 0.27		2.602079 (5.900016)			0.44		− 1.699185 (6.849454)			− 0.25	
DLnGDPpc2	0.0982585 (0.3279211)			0.30		− 0.1372083 (0.3364654)			− 0.41		0.1072338 (0.3896308)			0.28	
DLnOCpc	0.7703335*** (0.0749801)			10.27		0.7718326*** (0.0719638)			10.73		0.7519719*** (0.0838268)			8.97	
DLnNGaspc	0.05342* (0.0262725)			2.03		0.0680672*** (0.0123154)			5.53		0.0581397*** (0.0134327)			4.33	
DlnNGaspc(− 1)	− 0.0240029 (0.0208392)			− 1.15		–			–		–			–	
DLnHydropc	− 0.1221059** (0.0566155)			− 2.16		− 0.1398767*** (0.041813)			− 3.35		− 0.1129096*** (0.0353368)			− 3.20	
DLnRECpc	− 0.0071283 (0.0261782)			− 0.27		− 0.0026785 (0.0230384)			− 0.12		0.0191331 (0.0187674)			1.02	
DLnRECpc(− 1)	− 0.0052399 (0.0162018)			− 0.32		− 0.0197791 (0.0174509)			− 1.13		–			–	
DLnAgriland	–			–		0.7964744 (0.8968569)			0.89		− 0.0200886 (0.2039579)			− 0.10	
DLnTrade	–			–		–			–		0.0005463 (0.0264114)			0.02	
Constant	19.28221 (14.75453)			1.31		7.048558 (14.80404)			0.48		5.823078 (13.57771)			0.43	
Chile															
		Model 1 (1,2,2,1,2,2,2)					Model 2 (2,2,2,1,1,2,0,1)					Model 3 (1,1,0,1,1,0,0,1,0)			
ECT(− 1)		− 0.4256209*** (0.1273113)			− 3.34		− 0.707453*** (0.1442373)			− 4.90		− 0.7178329*** (0.1033298)			− 6.95
Long-run estimations															
LnGDPpc(− 1)		− 3.172742 (2.941803)			− 1.08		− 5.947781*** (1.754796)			− 3.39		− 3.997114** (1.490585)			− 2.68
LnGDPpc2(− 1)		0.1994798 (0.1570183)			1.27		0.365072*** (0.0960864)			3.80		0.2618448*** (0.0825639)			3.17
LnOCpc(− 1)		0.4714033*** (0.1185206)			3.98		0.404169*** (0.0574632)			7.03		0.3781112*** (0.0684468)			5.52
LnNGaspc(− 1)		0.0166914 (0.040481)			0.41		0.0230268 (0.0180607)			1.27		− 0.0013846 (0.0202744)			− 0.07
LnHydropc(− 1)		0.0068622 (0.1173394)			0.06		− 0.093132* (0.047123)			− 1.98		− 0.2521945*** (0.0396949)			− 6.35
LnRECpc(− 1)		0.0344374 (0.0255483)			1.35		− 0.023153* (0.0121484)			− 1.91		− 0.0378944** (0.0158016)			− 2.40
LnAgriland(− 1)		–			–		− 2.009403*** (0.4417916)			− 4.55		− 2.616017*** (0.5344704)			− 4.89
LnTrade(− 1)		–			–		–			–		0.1742773*** (0.0572525)			3.04
Short-run estimations															
DLnGDPpc		1.665535 (3.161661)			0.53		− 0.4958624 (2.467914)			− 0.20		− 2.585739** (1.073513)			− 2.41
DLnGDPpc(− 1)		− 9.483017*** (2.887545)			− 3.28		− 6.769085*** (2.245582)			− 3.01		–			–
DLnGDPpc2		− 0.0620437 (0.1806148)			− 0.34		0.0623216 (0.1417502)			0.44		0.1879608*** (0.0610787)			3.08
DLnGDPpc2(− 1)		0.5490947*** (0.1690161)			3.25		0.3978518*** (0.1316831)			3.02		–			–
DLnOCpc		0.5597824*** (0.0839201)			6.67		0.5756713*** (0.0686688)			8.38		0.5590349*** (0.0797917)			7.01
DLnNGaspc		0.1007175*** (0.0309688)			3.25		0.1051515*** (0.0233582)			4.50		0.0482629** (0.0235089)			2.05
DLnNGaspc(− 1)		0.0028841 (0.0261624)			0.11		–			–		–			–
DLnHydropc		− 0.0869725* (0.04503)			− 1.93		− 0.1149218*** (0.0294118)			− 3.91		− 0.1810336*** (0.0354578)			− 5.11
DlnHydropc(− 1)		− 0.1318324*** (0.0313572)			− 4.20		− 0.0931326*** (0.0328675)			− 2.83		–			–
DLnRECpc		− 0.0032152 (0.0168113)			− 0.19		− 0.0163797* (0.0087685)			− 1.87		− 0.0272019** (0.0109596)			− 2.48
DLnRECpc(− 1)		− 0.040201** (0.0187582)			− 2.14		–			–		–			–
DLnAgriland		–			–		− 0.1540564 (0.4099375)			− 0.38		− 0.680673 (0.4794734)			− 1.42
DLnTrade		–			–		–			–		0.125102*** (0.0383563)			3.26
Constant		− 2.098415 (5.553907)			− 0.38		10.20475* (5.602151)			1.82		5.874184 (5.428679)			1.08

ECT denotes error correction term; the prefix D denotes first difference and (− 1) indicates 1 lag

^*^, **, ***10%, 5%, and 1% statistical significance level, respectively

In the case of Argentina, we could confirm the validity of the EKC in the long run as the coefficients of LnGDPpc and LnGDPpc2 were positive and negative, respectively, in all three models we analyzed (${\theta }_{11}>0, {\theta }_{12}<0$ in model 1, ${\theta }_{21}>0, {\theta }_{22}<0$ in model 2, and ${\theta }_{31}>0, {\theta }_{32}<0$ in model 3, respectively).^7^ However, we could not find any evidence of the EKC in the short run. In relation to energy consumption, we found that LnOCpc and LnNGaspc contribute importantly to worsening environmental quality in all three models (the coefficients were statistically significant at 1% level) both in the short and long term, but in the case of natural gas, it had significantly lower positive impact on carbon dioxide emissions than oil. This finding is consistent with the previous empirical studies that energy consumption from fossil fuels accounts for the increase in CO_2_ emissions and natural gas, among the fossil fuel sources, emits less amounts of CO_2_ (Anser et al. 2020; Hanif 2017; Seri and Fernández 2021; Zambrano-Monserrate et al. 2018) so it might be a good alternative to replace other high-polluting intensive fossil fuel energy sources like oil and coal in the short run when economy shifts to low-carbon energy transition. Regarding LnRECpc, it had a significant impact on only model 1 in the long run. However, contrary to our belief, renewable energy consumption leads to an increase in CO_2_ emissions in Argentina although the magnitude of coefficient was very small (0.019). Regarding LnAgriland, it has a significant positive impact on carbon emissions in both the short and long run. In model 2, a 1% increase in agricultural land leads to an increase of CO_2_ emissions by about 0.52% and 0.46% in the short and long run, respectively, while in model 3, LnAgriland has short-term and long-term elasticities of 0.34% and 0.26%, respectively. Land use change, mainly due to the expansion of agricultural land and deforestation, is an important factor to be taken into account in terms of CO_2_ emissions in Argentina. This is because Argentinian economy relies heavily on commodity exports (soybeans, corn, and wheat among others) and CO_2_ emissions caused by land use change are expected to keep growing in the foreseeable future. Regarding the ECT, it has a negative sign and statistically significant at 1% level in all three models. This shows that there is a long-run equilibrium relationship among the variables we used in our regression models, which supports the results of cointegration test implemented before. Furthermore, the coefficient of ECT tells us about the speed of adjustment. In Argentina, all three estimated models were found to have a relatively high speed of adjustment (− 0.768 in model 1, − 0.879 in model 2, and − 0.790 in model 3). This means that disequilibrium caused by shock is corrected in the following period by about 76.8%, 87.9%, and 79% in models 1, 2, and 3, respectively.

- Brazil

In the case of Brazil, we found a U-shaped curve between economic growth and CO_2_ emissions in the long run (${\theta }_{11}<0, { \theta }_{12}>0$ in model 1, ${\theta }_{21}<0, {\theta }_{22}>0$ in model 2, and ${\theta }_{31}<0, {\theta }_{32}>0$ in model 3). This means that the level of carbon dioxide emissions initially decreases, but after a certain threshold, it increases again as Brazilian economy continues its growth. With respect to energy consumption, we found that fossil fuel energy sources, especially oil, influence significantly on deteriorating environmental quality since it leads to an increase of carbon dioxide emissions in the range of about 0.752% to 0.772% in the short run while in the long run it influences on increasing CO_2_ emissions in the range of about 0.779% to 0.846% when there is an increase of 1% in LnOCpc in three models. Regarding natural gas, it also leads to an increase in carbon dioxide emissions but to a lesser extent than oil since both the short and the long-term elasticities of LnNGaspc are significantly lower than those of LnOCpc (in the range of about 0.053% to 0.068% in the short run and in the range of about 0.065% to 0.076% in the long run). In relation to LnHydropc, it has a statistically significant negative impact on carbon dioxide emissions both in the short and long run in all three models. In addition, we observed that the negative impact of LnHydropc on LnCO_2_pc was stronger in the long term than the short term as absolute values of the long-term elasticities were systematically greater than those of the short-term elasticities in all three models. This finding suggests that the generation of electricity from hydro energy sources keeps playing a key role in the decarbonization of the Brazilian economy in the future. Regarding LnRECpc, we found that it only has a significant impact on LnCO_2_pc in the long run in model 2. However, contrary to our belief, the long-term elasticity of LnRECpc had a positive sign (0.026) which means that a 1% increase in LnRECpc leads to an increase of LnCO_2_pc by about 0.026%. This result suggests that consumption of non-conventional and new renewable sources (solar photovoltaic, wind, and biomass) still does not have a significant impact on the reduction in carbon dioxide emissions and more targeted energy policy is required to promote energy consumption from clean energy sources and to increase the share of renewables in energy mix in Brazil. Regarding LnAgriland and LnTrade, the estimation results tell us that they do not have significant impact neither in the short nor the long term in three models. Lastly, the signs of ECT were negative and statistically significant at 1% level in all models supporting the previous cointegration bounds test results. Also, we observed a high speed of adjustment in each model (− 0.916, − 0.991, and − 0.888 in models 1, 2, and 3, respectively).

- Chile

In the case of Chile, we found a U-shaped curve relationship between economic growth and carbon dioxide emissions in models 2 and 3 in the short and long run (particularly in model 2, we observed a U-shaped curve in lagged first differenced of LnGDPpc and LnGDPpc2). Regarding energy consumption, LnOCpc exerts a positive effect on carbon dioxide emissions both in the short and long run. However, we noticed that the elasticities of LnOCpc were systematically lower in comparison with Argentina and Brazil (especially for long-term elasticities of LnOCpc). In other words, the negative impact of oil consumption on environmental quality in terms of CO_2_ emissions in Chile is less than that of Argentina and Brazil. Regarding LnNGaspc, it has a significant positive impact on LnCO_2_pc only in the short run. With relation to LnHydropc, it has a negative impact on carbon emissions in models 2 and 3 in the long run (− 0.093% and − 0.252% in LnCO_2_pc at 10% and 1% significance level, respectively, when there is a 1% increase in LnHydropc) while in the short run it leads to a reduction in carbon emissions by about 0.087%, 0.115%, and 0.181% in models 1, 2, and 3 with a statistical significance level of 10%, 1%, and 1%, respectively, when LnHydropc increases by about 1%. With regard to LnRECpc, it has a negative sign and statistically significant at least 10% level in models 2 and 3 both in the short and long run (in model 1, DLnRECpc(− 1) has a significant negative impact at 5% significance level). It is worth noting that Chile is the only one among the three countries in our investigation where renewable energy consumption has a significant negative impact on carbon dioxide emissions although the absolute value of its elasticities is relatively small in comparison with that of fossil fuels (especially with LnOCpc). Therefore, it is important that the Chilean government implements adequate energy policy such that it allows to keep promoting and providing incentives among the citizens in the use of renewable energy. Moreover, government should make efforts to facilitate the financing of large-scale renewable energy deployment and the integration of renewable energy sources in energy mix in short and medium term to fully enjoy its benefits in the long term. Regarding LnAgriland, we found an unusual large negative impact on CO_2_ emissions in the long run. The estimation results tell us that a 1% increase in LnAgriland leads to a reduction in LnCO_2_pc by about 2.009% and 2.616% in models 2 and 3, respectively. If we compare the impact of LnAgriland on carbon emissions in Chile with that in Argentina and Brazil, the difference between them is very clear (in Argentina, LnAgriland contributes to increasing CO_2_ emissions in the short and long run while in Brazil it does not have a significant impact). This result may suggest sustainable agricultural practices carried out in Chile in comparison with Argentina or Brazil during the period of our study. Regarding LnTrade, it leads to an increase of LnCO_2_pc by about 0.125% and 0.174% in the short and long run, respectively, when there is a 1% increase in LnTrade. Lastly, ECT(− 1) tells us that there is a long-run equilibrium relationship among the variables in our regression models since it has a negative sign and statistically significant at 1% level. Moreover, we could observe that the speed of adjustment of three models in Chile is relatively slower if we compare ECT(− 1) of Argentina and Brazil meaning that, when the shock takes place, the Chilean economy returns to its equilibrium level at slower pace than Argentinian or Brazilian case.

### Postestimation tests

After the estimation of our models by using ARDL-ECM technique, we carried out postestimation tests to see whether the models estimated are well behaved (Menegaki, 2020). We used the Jarque–Bera test to check the normal distribution, the Breusch-Pagan test and the White test to check heteroskedasticity, the Breusch-Godfrey serial correlation LM test and the Durbin-Watson test to check autocorrelation, and lastly the Ramsey RESET test to see whether functional forms of our regression models are well specified or not (Menegaki, 2020; Shahbaz et al. 2012).

In Table 6, we can observe that in general, our regression models are well behaved and do not suffer from heteroskedasticity, serial correlation, and omitted variable bias. However, the assumption of the normal distribution of the residuals was not verified regardless of the countries and the EKC models estimated as the null hypothesis of the Jarque–Bera test was rejected in all cases. This may be due to our small sample size (44 observations for each country) as the Jarque–Bera test is normally used for large sample (more than 100 observation) and it does not perform well in the case of small samples. Apart from the results of no normality obtained in our models, the Breusch-Pagan test points out that our three estimated EKC models might have heteroskedasticity issue in the case of Brazil, but two other tests (the White test and the ARCH test) indicate no heteroskedasticity problem in our models. In the case of Chile, the Ramsey RESET test indicates that models 1 and 2 might suffer from omitted variable bias.

**Table 6:** Postestimation test results

Test type	Model 1		Model 2		Model 3	
	${\chi }^{2}$	Probability	${\chi }^{2}$	Probability	${\chi }^{2}$	Probability
Argentina						
Jarque–Bera test	7.22	0.0271	23.58	0.0000	12.27	0.0022
Breusch-Pagan test	0.22	0.6383	0.04	0.8374	0.40	0.5267
White test	43.00	0.4282	43.00	0.4282	43.00	0.4282
ARCH test	0.185	0.6672	0.451	0.5017	0.238	0.6256
Breusch-Godfrey LM test	0.551	0.4578	0.640	0.4238	0.708	0.4001
Durbin-Watson	0.364	0.5465	0.393	0.5309	0.402	0.5262
Ramsey RESET test	1.12	0.3581	0.66	0.5821	0.86	0.4757
Brazil						
Jarque–Bera test	4.78	0.0917	6.91	0.0316	7.51	0.0234
Breusch-Pagan test	3.88	0.0489	4.88	0.0272	4.90	0.0269
White test	43.00	0.4282	43.00	0.4282	43.00	0.4282
ARCH test	1.157	0.2822	1.057	0.3040	0.763	0.3825
Breusch-Godfrey LM test	2.116	0.1458	2.229	0.1354	1.550	0.2131
Durbin-Watson	1.499	0.2287	1.422	0.2331	0.898	0.3434
Ramsey RESET test	1.74	0.1834	1.422	0.2331	1.91	0.1574
Chile						
Jarque–Bera test	6.56	0.0377	71.28	0.0000	–	0.0000
Breusch-Pagan test	0.91	0.3409	0.83	0.3612	0.54	0.4630
White test	43.00	0.4282	43.00	0.4282	43.00	0.4282
ARCH test	2.320	0.1277	0.223	0.6368	0.018	0.8935
Breusch-Godfrey LM test	0.103	0.7484	0.026	0.8726	0.878	0.3488
Durbin-Watson	0.067	0.7956	0.016	0.9008	0.500	0.4794
Ramsey RESET test	4.78	0.0088	3.88	0.0216	1.12	0.3635

Jarque–Bera test H_0_: normal distribution of the residuals; Breusch-Pagan test: H_0_: homoskedasticity; White test: H_0_: homoskedasticity; ARCH test: H_0_: no ARCH effects; Breusch-Godfrey test: H_0_: no serial correlation; Durbin-Watson test: H_0_: no serial correlation; Ramsey RESET test: model has no omitted variables

### CUSUM and CUSUMSQ tests

After postestimation tests, we examined the stability of our models by using CUSUM and CUSUMSQ tests. These tests are used to support the estimation results obtained previously in ARDL-ECM by predicting the stability of the long-run relationship among the variables and to detect structural breaks in our regression models (Menegaki, 2020). For each country and model, the CUSUM tests are presented on the left-hand side while on the right-hand side the CUSUMSQ tests are presented.

**Fig. 2:**
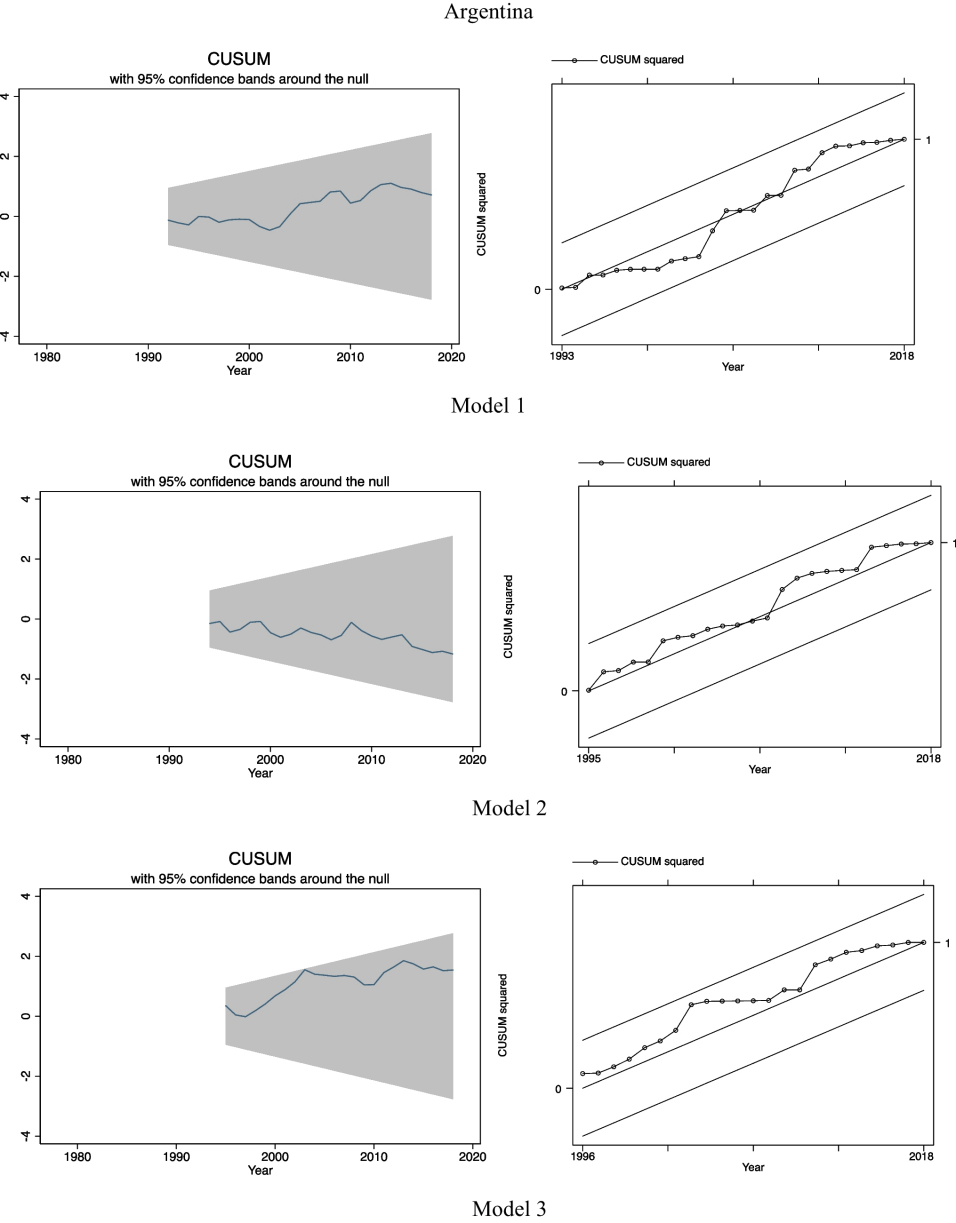

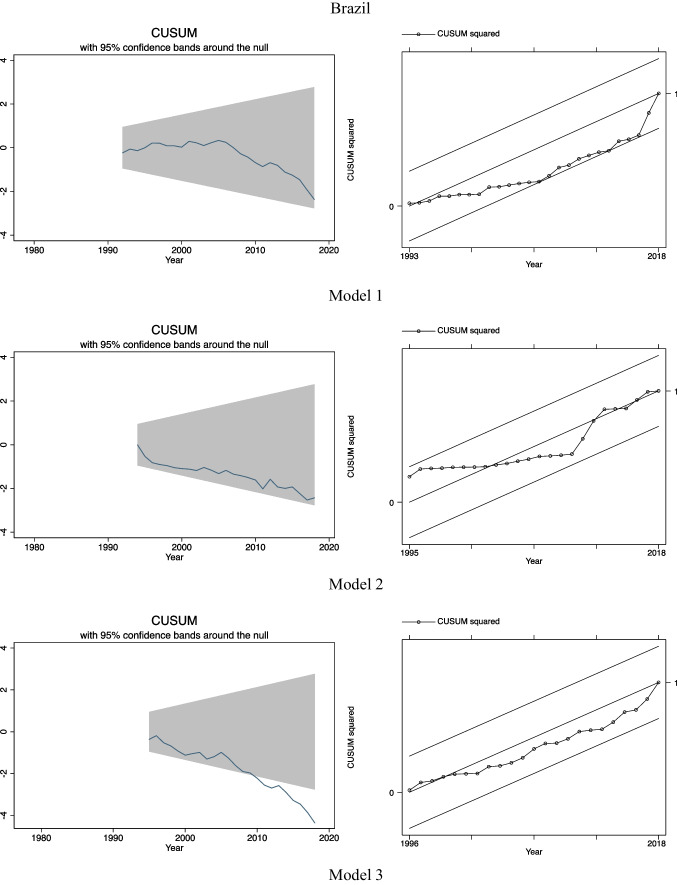

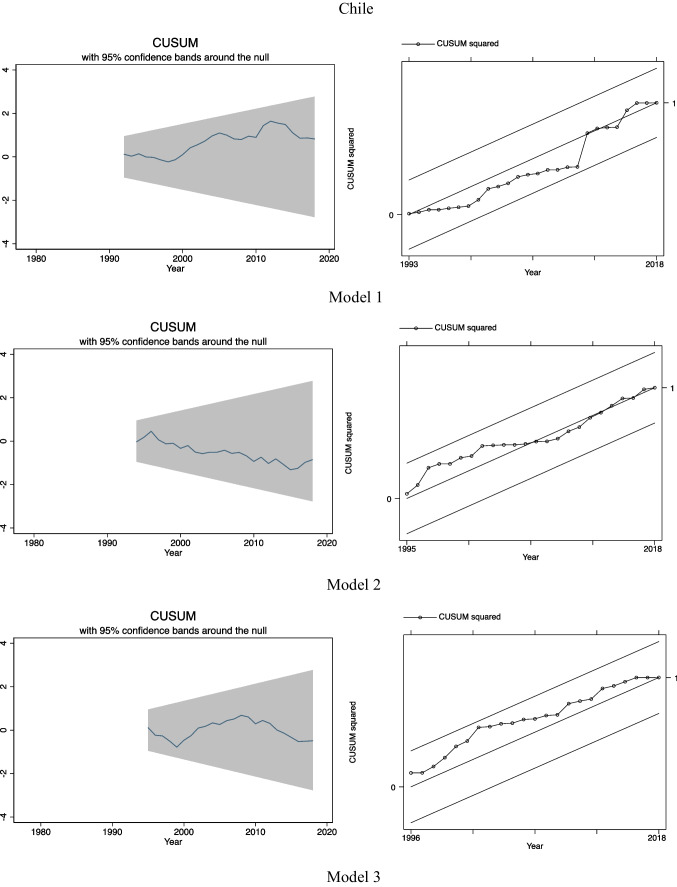
CUSUM and CUSUMSQ tests for 3 estimated models for Argentina, Brazil, and Chile

As we can see in Fig. 2, in general terms, the assumption of the stability of our models (in other words, the stability of the long-run equilibrium relationships among the variables) is confirmed according to the CUSUM and the CUSUMSQ tests as all the lines lie within the 95% confidence bands. Only in the case of Brazil, we observed some instability after the end of 2000s. According to the CUSUM test for model 3, we observed that the line keeps maintaining within the 95% confidence bands up to the end of 2000s, but after then, it deviates from them. However, according to the CUSUMSQ test for model 3 in Brazil, there is no evidence of instability over time as the line always remains within the 95% confidence bands.

### The Toda-Yamamoto Granger causality test

After having confirmed the cointegration relationships among the variables by means of cointegration bounds test and having estimated the short- and long-run effects by using the ARDL-ECM method, the last step of our analysis consists in determining the direction of causality between the variables with the help of the Toda-Yamamoto Granger causality test. The advantage of using this test is that it can be used regardless of stationarity of the variables, so we do not need to transform variables into the first differences in case the variables are not stationary at levels. In the Toda-Yamamoto Granger causality test, we exclusively centered our attentions to two different directions of causality relationships: on the one hand, the direction of causality between LnCO_2_ and independent variables to see whether it supports our previous ARDL-ECM estimation results obtained in previous stage. Otherwise, the direction of causality between economic growth and four different sources of energy consumption (oil, natural gas, hydro, and renewable energy) was examined.^8^ Furthermore, for the sake of simplicity and to avoid redundancy matter, we excluded the variable LnGDPpc2.

As we can see in Table 7, in the case of Argentina, LnCO_2_pc was Granger caused by economic growth (proxied by LnGDPpc), LnOCpc, LnNGaspc, and LnHydropc. We also found a bidirectional relationship between LnAgriland and LnCO_2_pc. This finding supports our previous estimation results that both economic growth and energy consumption contribute importantly to carbon dioxide emissions. Regarding the direction of causality between economic growth and energy consumption, both LnOCpc and LnNGaspc are Granger caused by economic growth (conservation hypothesis) while in the case of LnHydropc and LnRECpc growth hypothesis was being fulfilled, namely, country’s income level determines energy consumption from hydroelectricity and renewable energy. In the case of Brazil, we found a unidirectional causality running from LnGDPpc, LnRECpc, LnAgriland, and LnTrade to LnCO_2_pc. It is worth noting that we could not find any evidence of the causality relationships between LnOCpc and LnHydropc with LnCO_2_pc which is quite surprising since according to our previous ARDL-ECM estimation both oil and hydroelectricity consumption had a significant impact on carbon dioxide emissions in both the short and long term. In relation to the nexus energy consumption-economic growth, the conservation hypothesis was met for LnOCpc and LnRECpc; in other words, there is a unidirectional causality running from per capita oil consumption and per capita renewable energy consumption to economic growth. Besides, we observed that LnAgriland had a bidirectional causality relationship with economic growth. In the case of Chile, LnCO_2_pc is Granger caused by LnHydropc and LnRECpc which is consistent with our ARDL-ECM estimation results obtained previously. However, we could not verify the causality between economic growth and LnOCpc with LnCO_2_ as in previous ARDL-ECM estimation. In relation to economic growth, we found that LnAgriland, LnNGaspc, LnHydropc, and LnRECpc Granger cause LnGDPpc. In the case of the latter, bidirectional causality relationship was also confirmed. Lastly, we could observe conservation hypothesis between LnGDPpc and LnOCpc, in other words, a unidirectional causality running from economic growth to oil consumption.

**Table 7:** Toda-Yamamoto Granger causality tests

Dependent variables	Independent variables												
	LnCO2pc	LnGDPpc	LnOCpc		LnNGaspc		LnHydropc	LnRECpc			LnAgriland		LnTrade
Argentina													
LnCO2pc		9.0255***	3.6549*		5.8967**		7.7752***	0.04759			17.966***		2.4681
LnGDPpc	0.49322		2.1916		1.2297		3.1693*	1.8638			12.198***		3.2532*
LnOCpc	2.4295	8.2442***			12.793***		15.419	1.8228			21.308***		0.63087
LnNGaspc	1.0587	0.81457	0.03135				6.8343**	0.56967			3.9323*		0.27651
LnHydropc	0.20433	0.09403	0.50393		1.3784			0.01103			5.3906**		1.2688
LnRECpc	0.32114	13.601***	0.00244		0.23852		0.07888				0.15352		3.8933*
LnAgriland	3.0584*	0.05929	5.459**		9.1268***		0.01234	5.6169**					11.76***
LnTrade	2.424	6.1043**	5.5735**		4.125*		0.02524	6.8397**			9.8483***		
Brazil													
LnCO2pc		18.499***	0.01127		0.53143		0.98882	5.266**			36.772***		6.7633**
LnGDPpc	0.06972		0.22684		1.3039		0.9964	1.1995			3.2628*		2.7609
LnOCpc	0.36875	17.48***			1.3186		0.36437	11.754***			66.063***		5.3085**
LnNGaspc	1.8212	0.20243	4.1783**				12.411***	0.5267			0.09322		7.2561**
LnHydropc	0.4398	0.41541	0.00459		2.247			0.15674			2.1722		1.459
LnRECpc	0.34255	11.989***	1.1815		0.07135		0.05804				0.03668		4.2289**
LnAgriland	0.54536	25.665***	1.7789		2.7164		0.27466	6.7923**					7.0572**
LnTrade	0.04238	0.39973	1.6137		0.00987		5.6364**	3.632*			0.95133		
Chile													
LnCO2pc		2.117	0.00055	0.0378		8.8533***			10.019***	2.2279		1.8717	
LnGDPpc	6.8241**		0.25374	3.3233*		6.8472**			22.732***	10.022***		2.9824*	
LnOCpc	2.4088	7.693***		4.7518**		7.0404**			11.496***	6.5963**		5.1975**	
LnNGaspc	4.675**	0.64943	2.1656			1.0267			1.7761	18.48***		0.04457	
LnHydropc	0.10475	0.23825	0.00223	17.499***					1.4058	1.6161		0.40972	
LnRECpc	0.19343	5.6058**	9.7184***	7.3274**		1.1283				1.3165		14.764***	
LnAgriland	1.4726	2.1529	1.6711	3.4541*		1.5467			7.6397***			0.11461	
LnTrade	0.14316	0.00192	0.97562	1.6967		6.7398**			0.93991	0.01029			

Before we proceed to examine the Toda-Yamamoto Granger causality test, we determined optimal lags and estimated VAR regression by using “varsoc” and “var” command, respectively, in Stata. The optimal lags chosen for Argentina, Brazil, and Chile were 3, 4, and 3, respectively

^*^, **, ***10%, 5%, and 1% statistical significance level, respectively

## Conclusion and policy implications

To avoid catastrophic consequences of global warming, more efforts should be made to reduce carbon dioxide emissions known as the major factor precipitating global warming. For developing middle-income economies such as LAC countries, the energy demand and the use of natural resources are expected to be highly demanded in the process of their economic growth, which stimulates increase in carbon emissions and waste leading to environmental degradation. To alleviate the negative impacts of economic growth on environmental degradation and achieve a climate resilient, inclusive economic growth in the LAC region, a transition from polluting energy sources to clean energy sources might be a good strategy to achieve the goals mentioned above. In this study, we thoroughly examined the impacts of economic growth, consumption of four different types of energy sources, namely, oil, natural gas, hydroelectricity, and unconventional renewable energy (solar, wind, geothermal, and bioenergy), in addition to agricultural land and trade openness as control variables on environmental quality in the framework of the EKC for a set of 44 year time series data (1975–2018) of 3 LAC countries: Argentina, Brazil, and Chile based on the recognition of the importance decoupling economic growth from environmental degradation and of the critical role energy consumption patterns play in the evolution of the economy in terms of carbon dioxide emissions.

According to the estimation results of ARDL-ECM, we only could verify the EKC hypothesis (inverted U-shaped curve) in Argentina in the long run but not in the others, Brazil and Chile, where the U-shaped curve relationship was found between economic growth and CO_2_ emissions. This result suggests that the evidence of the EKC is not robust in these three economies, and especially in the case of Brazil and Chile, the careful attention should be paid to monitor the evolution of CO_2_ emissions in the long run since it also shows an increasing trend as the economy grows after having reached a certain threshold level of income. In relation to the impacts of consumption of four different types of energy on environmental degradation, fossil fuel sources, especially oil, were found to have a strong positive impact on CO_2_ emissions in Argentina, Brazil, and Chile in both the short and long run. As for natural gas, its consumption leads to an increase in CO_2_ emissions in three countries together (in Chile, the consumption of natural gas has a significant impact on CO_2_ emissions only in the short run) but it has a significantly lower impact on environmental degradation than oil consumption as both the short and long-term elasticities of consumption of natural gas with respect to CO_2_ emissions are quite lower than those of oil in absolute values. This finding implies that natural gas might be served as an intermediate backup energy source good enough in the short and medium term on the way to their low-carbon energy transition (shift from dirty energy sources to clean renewable energy sources) which ultimately aims at reaching net zero carbon emissions target by 2050 documented in the Paris Agreement in 2015. That is because natural gas emits significantly less amounts of CO_2_ in comparison with other fossil fuels such as coal and oil and it can support and complement renewable energy sources as backup energy even when intermittence issue is present. However, careful attention should be made by governments to avoid potential lock-in fossil fuel-based technologies and path dependence in the long run as use of natural gas might discourage investments in renewable energy deployment and could become a stumbling block to innovation in clean energy technology. Regarding hydroelectricity consumption, it contributes to reducing CO_2_ emissions in Brazil and Chile in both the short and long run while in Argentina its meaningful impact is not manifested (in Chile, the hydroelectricity consumption has a significant negative impact on CO_2_ emissions in the long run only for models 2 and 3, namely, when two control variables were added in our regression analysis). This finding emphasizes that hydropower will continue to play an important role in reducing CO_2_ emissions in Brazil and Chile in the future and also combining with another clean energy sources such as the renewables could help to enhance energy security in terms of diversification of energy mix and responding to growing energy demands caused by population growth and economic activities in these economies. With respect to renewable energy consumption, only in Chile, we could verify a significant negative impact on CO_2_ emissions in models 2 and 3 in the short and long run (although the elasticities in absolute value were significantly lower than those of oil or natural gas which means that consumption from renewable energy sources was shown to be not sufficient to compensate the negative impact on environmental quality driven by fossil fuels) while in Argentina and Brazil renewable energy consumption had a significant impact only in the long run (in model 1 for Argentina and in model 2 for Brazil, respectively), but unlike our expectation, it had a positive sign, so in this regard, significant efforts should be made by governments to increase the share of renewables in the primary energy consumption in their economies. It would be a challenging issue for governments to find feasible approaches to achieving the goals. One possible approach we suggest is to increase awareness to the benefits of using renewable energy sources among consumers (not only in environmental aspect but also in social and economic terms as well). Another approach we suggest is based on making customers change their behaviors in using energy sources through carbon pricing and gradual elimination of subsidies to fossil fuels which are already high in these economies (the revenues obtained from the former can be used to provide more targeted support to low-income households affected by the latter).

Regarding the direction of causalities between economic growth and energy consumption, the Toda-Yamamoto Granger causality test tells us that there is a unidirectional causality running from LnHydropc to LnGDPpc (growth hypothesis) while for the opposite direction, namely, a unidirectional causality running from economic growth to energy consumption was found in LnOCpc and LnRECpc, respectively (conservation hypothesis) in Argentina. In Brazil, we could verify the conservation hypothesis in LnOCpc and LnRECpc. In Chile, we found that conservation hypothesis held for LnOCpc while the growth hypothesis was found to be valid for LnNGaspc, LnHydropc, and LnRECpc. These findings tell that oil consumption can be drastically reduced and replaced by other clean energy sources with significantly less CO_2_ emissions along with their economic growth in Argentina, Brazil, and Chile on the basis of the conservation hypothesis saying that the reduction in oil consumption does not hinder economic growth in these countries. Furthermore, our findings recommend that energy consumption from natural gas, hydropower, and renewables leads to economic growth in Argentina and Chile (these 3 types of energy all granger caused economic growth in Chile while only hydropower did in the case of Argentina); thus, increased energy consumption of these 3 energy sources might help to boost economic growth along with achieving environmental protection goal in Argentina and Chile.

In our study, we considered renewable energy consumption in its entirety without specific considerations on types of energy sources in separate: solar, wind, geothermal, and bioenergy. For further investigation, more specific and sophisticated approaches are required to clarify the effects of specific renewable energy sources on carbon emissions and their nexus with economic growth in LAC countries. Also, more studies are expected to investigate the impacts of adopting abatement technologies such as Carbon Capturing Utilization and Storage (CCUS) and digital technologies and their potential synergistic impacts with renewable energy consumption on reducing CO_2_ emissions and boosting economic growth in the LAC region.

## Data Availability

Not applicable.

## Footnotes

1 According to IRENA, the renewables’ share in electricity generation in South America and Central America and Caribbean in 2015 was 64.2% and 29.4%, respectively, while the world average was just only 22.8%.

2 According to the World Meteorological Organization (WMO 2021), in the LAC region, more than 27% of the population live in coastal areas where 6–8% of them are exposed to high or very high risk of suffering coastal hazards. This is especially true for low-lying Caribbean states.

3 According to the World Bank Data in 2020, Brazil was ranked first in terms of GDP at constant 2015 US dollars while Argentina and Chile were ranked third and fifth, respectively.

4 According to the World Bank Data in 2020, Brazil, Argentina, and Chile were ranked the second, third, and fifth largest emitters of CO_2_ emissions measured in kilotons, respectively.

5 In case of land use change, unsustainable expansion of agricultural lands by deforestation reduces nature’s capacities of absorbing CO_2_ emissions which leads to an increase in environmental degradation while in case of trade openness its impact on CO_2_ emissions is far from clear. On the one hand, it might have a positive impact on environmental quality through the import of clean energy technology from developed countries. Otherwise, it might lead to increasing CO_2_ emissions via export activities in case of increased energy consumption required to produce more goods or services mainly comes from dirty energy sources.

6 Excluding LAC countries.

7 See Eqs. (7), (8), and (9).

8 Regarding the nexus between energy consumption and economic growth, there are 4 well-known types of hypotheses, namely, growth hypothesis, conservation hypothesis, neutrality hypothesis, and feedback hypothesis. In growth hypothesis, there is a unidirectional causality from energy consumption to economic growth while in conservation hypothesis the causality is reversed. Neutrality hypothesis refers to the absence of relationship between energy consumption and economic growth while in feedback hypothesis there is a bidirectional causality relationship between energy consumption and economic growth (Fuinhas et al. 2021).
